# Constructing a student development model for undergraduate vocational universities in China using the Fuzzy Delphi Method and Analytic Hierarchy Process

**DOI:** 10.1371/journal.pone.0301017

**Published:** 2024-03-22

**Authors:** Qiaona Xing, Huey Pyng Tan, Su Wan Gan

**Affiliations:** 1 Asean Research Center, Dezhou University, Dezhou, Shandong, China; 2 Faculty of Arts and Social Science, Universiti Tunku Abdul Rahman, Kampar, Perak, Malaysia; National University of Sciences and Technology, PAKISTAN

## Abstract

As the industrial structure changes, the severe shortage of high-quality technical and skilled talent in China is one of the most significant factors affecting the high-quality development of China’s economy. Bridging the gap between cultivating talent from new undergraduate vocational universities and the demand for industrial talent is regarded as an efficient strategy to address the talent shortage. In addressing the gap, China is hindered by a lack of clarity regarding student development goals and effective assessment instruments. Thus, this study aimed to use the Fuzzy Delphi Method (FDM) and the Analytical Hierarchy Process (AHP) to overcome the above challenges. Specifically, we used the FDM to establish a five-level undergraduate vocational education student development model with two 2^nd^-level indicators, three 3^rd^-level indicators, eight 4^th^-level indicators, and 33 5^th^-level indicators to clarify student development goals. Then, the AHP was applied to determine the indicator weights, and a student development assessment instrument was developed to help universities acquire student development data and improve the matching degree between talent supply and demand. This study could help undergraduate vocational universities cultivate high-quality technical and skilled talent quickly to meet the demand for China’s new economic system and to promote industry independence and global competitiveness.

## 1. Introduction

China urgently needs high-quality technical and skilled talent, due to the constant acceleration of industrialization and the emergence and development of new high-tech industries. In response to these talent requirements, China has established 32 undergraduate vocational universities since 2019 to cultivate high-quality technical and skilled talent. China’s Ministry of Education also declared that it would further encourage and develop them, with the aim of increasing the number of their students to no less than 10% of higher vocational education students by 2025. Now, undergraduate vocational universities are responsible for cultivating high-quality technical and skilled talent and promoting the high-quality development of China’s economy.

Improving the match between students’ abilities and the requirements for high-quality technical and skilled talent is the key to the effective development of undergraduate vocational universities. In recent years, the Ministry of Education, universities, and academics have paid close attention to the high-quality development of undergraduate vocational education. However, data on the ability structure and current development level of undergraduate vocational students remain limited [1]. Relevant policies have been established, but no clear and explicit criteria concerning the concept or student development goals exist. The existing theoretical achievements only focus on connotations, distinctions from other applied talent, development dilemmas, and principles of talent cultivation goals. Still, almost no research has been conducted on the talent that undergraduate vocational education needs to cultivate, its student development model, or the assessment instruments employed. The lack of clarity in goal orientation severely restricts the sustainability of undergraduate vocational education development in the future.

To tackle the above problems, it is imperative to establish a clear, pertinent, and actual student development goal (model) and to develop an effective student development assessment instrument for undergraduate vocational universities. In this study, we applied the Fuzzy Delphi Method (FDM) and the Analytical Hierarchy Process (AHP) to develop a model and assessment instrument for student development at undergraduate vocational universities. In particular, we focus on two issues: First, what levels, dimensions, and indicators should be included in the student development model? Second, what are the differences between the weights of student development indicators at different levels in the assessment instrument? This study explored the above issues to highlight the goal and direction and provide instrumental support for undergraduate vocational universities to cultivate high-quality technical and skilled talent.

## 2. Literature review

### 2.1 Undergraduate vocational education

Undergraduate vocational education belongs to the undergraduate level of higher education and is typologically subordinate to vocational education. It is characterized by higher education, vocational training, and local features [1]. It has the fundamental characteristics of higher education, while the vocational training aspect means that it focuses on enhancing students’ vocational techniques and abilities to make them more employable. Local features refer to its talent cultivation dovetailing with local industry development and enterprise production to boost local economies. Based on these features and previous research [1–3], in this study, undergraduate vocational education is defined as vocational education that cultivates high-quality technical and skilled talent by providing students with adequate professional theoretical knowledge, the ability to lead practical and technological applications and on-site handling, and transferable and innovative abilities that enable them to adapt to the needs of multiple industries.

Talent cultivation objectives are the provisions made by the cultivation unit for the educator’s cultivation direction, requirement specification, etc., based on educational goals and social needs [2]. In recent years, the literature on talent cultivation in undergraduate vocational education has focused on distinguishing its objectives from those of college vocational education and general undergraduate education, specifically in terms of “level” and “type” dimensions [4]. Undergraduate vocational education cultivates students with more profound technical and theoretical knowledge, more advanced practical skills, and a certain level of management, job conversion, technology transfer, and innovation abilities compared to colleges. Meanwhile, general undergraduate education cultivates academic talent with theoretical knowledge, professional ability, and moral quality among the three categories. In conclusion, academics have not reached a consensus regarding the details of talent cultivation in undergraduate vocational education.

### 2.2 Student development

College student development refers to the concept of human development within the context of higher education. Bowen (1977) argued that student development should encompass cognitive learning, practical abilities, and emotional and moral development [5]. Astin (1993) measured student development in terms of cognitive aspects (i.e., knowledge and ability) and emotional gains (i.e., students’ perceptions, attitudes, and values) [6]. According to Pang Bo (2012), student development in higher education involves five components: knowledge, ability, social relationships, quality, and personality [7]. He Xiangling regards student development as the holistic development of their knowledge, abilities, values, and interpersonal relationships in higher education [8]. This study defines student development as college students’ ongoing progress and improvement in their knowledge, abilities, and comprehensive qualities, representing the added value of college students’ four years of enrollment before graduation.

Scholars generally agree that assessing student development involves evaluating a university’s ability to cultivate certain qualities. Student development assessment is in a constant state of exploration worldwide. The United States was the first country to develop student development measurement instruments, including the famous College Student Experiences Questionnaire (CSEQ), developed in 1994, and the National Survey of Student Engagement (NSSE), published in 2000. In the CSEQ, student development covers general education, personal development, scientific and technological knowledge, and professional and practical abilities. The NSSE evaluates student development in terms of three dimensions: knowledge, ability, and value gains.

Student development assessment instruments in China are derived from the adaptation of traditional measurement instruments from other nations to the Chinese context. Representative instruments in China include the Ten Years of Student Development in Shanghai biennial research project (1998–2007), the State of Student Development in Beijing annual survey project (2006), and the Tracking Study of Learning and Development of Chinese University Students (CCSS, 2007). The CCSS is China’s most popular and longest-running assessment instrument for student learning engagement. The above instruments can be used to evaluate student development, learning engagement, and factors influencing student development [8]. However, they only apply to general undergraduate universities, vocational colleges, and Beijing’s prestigious universities.

### 2.3 FMD and AHP

In traditional Multiple Criteria Decision-Making, where rating criteria are accurately known, scholars often use the Delphi method to predict the results. The Delphi method anonymously solicits experts’ opinions in multiple rounds. They are repeatedly solicited, summarized, modified, and technically processed to obtain a consensus among experts and ultimately predict indicators.

However, criteria cannot be expressed under many conditions in terms of accurate data [9, 10]. Fuzzy theory has been proposed to cope with this limitation. Many scholars use the FDM to screen the indicators when researching evaluation indicator systems or models [11–14]. The FDM uses statistical analyses and fuzzy calculations to transform experts’ subjective opinions into quasi-objective data. It considers the uncertainty and fuzzy nature of experts’ subjective thinking for further screening indicators.

The AHP is often used to determine the importance of indicators [15–18]. It is a decision-making method combining qualitative and quantitative analyses by decomposing the problem into related factors and levels, such as objectives, criteria, and options. It is characterized by the hierarchical and quantitative nature of the decision-making process, which is based on the laws of thinking and psychology. It is widely used to solve complex decision-making problems to determine indicator weights.

Many researchers combine the FDM and AHP to enhance their findings (Table 1) [19–21]. This combination not only allows the expert’s opinion to be used as a decision-making parameter to screen indicators that express a particular item, but it also utilizes the expert’s two-by-two judgment of indicators to determine each indicator’s importance for the whole to determine a complete model that can express the item.

**Table 1:** FDM, AHP, and hybrid studies on the indicator system.

Author	Application area	Methods used
Ocampo (2018)	Selection of the most effective indicators of sustainable ecotourism	FMD
Sadeghi (2021)	Identification of the main risks associated with earthquakes in dilapidated urban textures	FMD
Etu (2022)	Constructing indicators affecting emergency department performance during a medical boom	FMD
Padilla-Rivera et al. (2021)	Identification of social circular economy indicators	FMD
Liu & Ning (2020)	Refining essential indicators for evaluating teachers’ practical exercises in enterprises	AHP
Panchal & Shrivastav (2022)	Assigning weights to different causal factors of landslide hazard on India’s national highways	AHP
Shen & Wang (2022)	Proving the contribution values and gradient values of floral organs	AHP
Tempa (2022)	Assessing flood vulnerability to floods in Bhutan	AHP
Chen (2018)	Developing indicators for sustainable campuses	Hybrid
Karam et al. (2021)	Analysis of the barriers to implementing horizontal collaborative transport	Hybrid
Li & Dong (2019)	Establishing a model for predicting the remaining service life of residential buildings	Hybrid

In summary, this study proposed a combination of the FDM and AHP to construct a student development model. The student development model concretizes student development goals by layering student development indicators according to student development theory. It can provide a realistic reference for undergraduate vocational universities to improve the matching of talent supply and demand.

### 2.4 Research gap

The literature review shows that academics and professionals alike are interested in undergraduate vocational education and student development. In the previous sections, this study features the following research gaps:

Section 2.1 highlights the lack of specific talent development objectives in undergraduate vocational education. Student development is the manifestation of talent cultivation. Consequently, student development model indicators also concretize talent cultivation objectives. It is essential to clarify the student development model indicators as undergraduate vocational education is responsible for cultivating talent for China’s high-quality economic and social development.

Section 2.2 identifies the research gap in student development assessment instruments for undergraduate vocational universities. Universities need instruments to assess the achievement of student development goals. They optimize student development programs based on assessment results to maximize the match between student development and social talent requirements.

Section 2.3 confirms the wide applicability of the FDM and AHP in model and indicator system research. To the best of our knowledge, no previous work has analyzed student development combining the FDM and AHP.

## 3. Methodology

**Fig 1:**
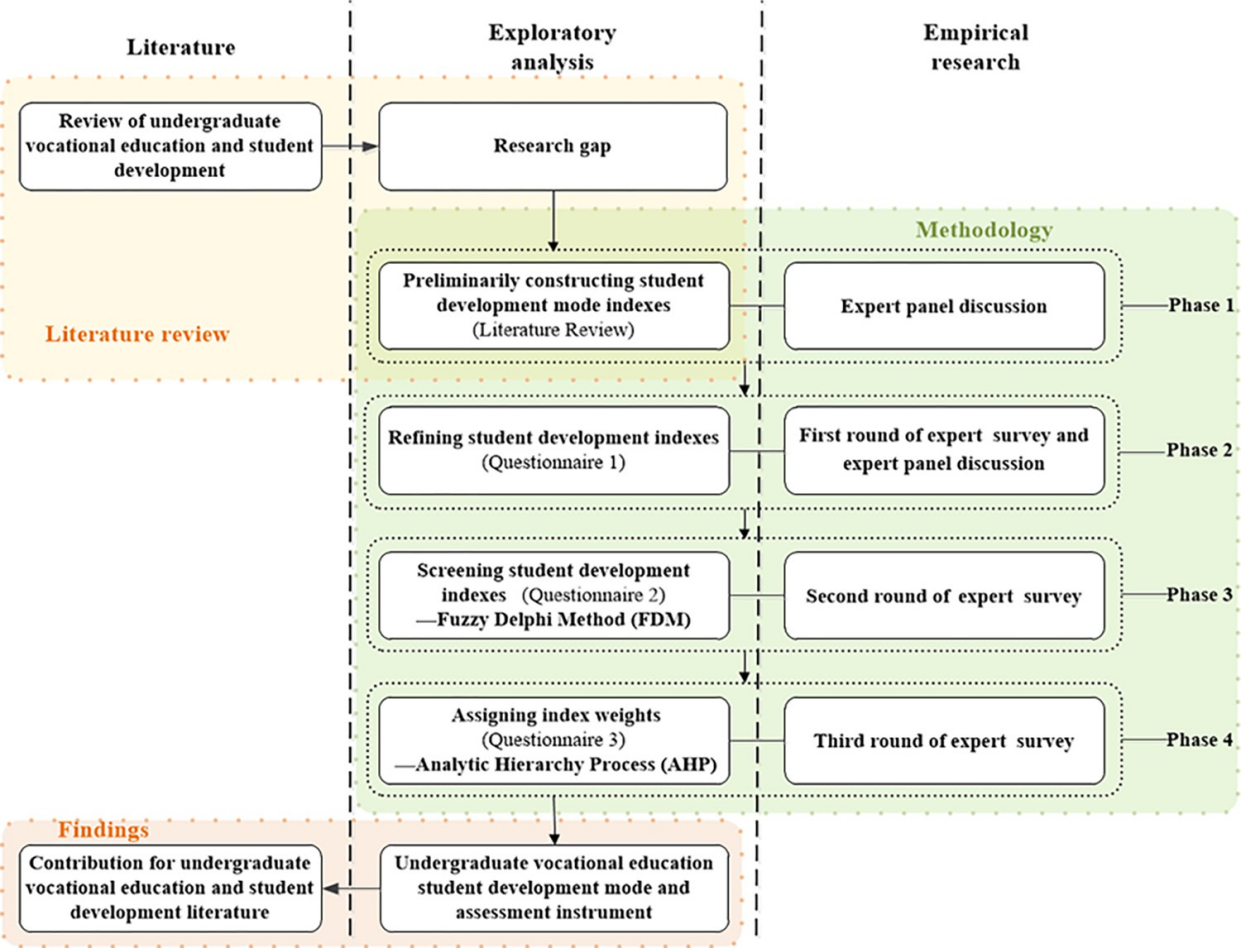
Research framework.

This study proposed a four-stage methodology for developing a model and assessment instrument for student development in undergraduate vocational education, including preliminary construction, refinement, screening, and weight determination indicators. Fig 1 shows the detailed research framework for this study, and the following sections explain each stage. Prior to the data collection, this study obtained a written approval from the Universiti Tunku Abdul Rahman Scientific and Ethical Review Committee to support collecting research data. Written informed consent was obtained from all participants for inclusion in the study.

### 3.1 Stage 1: Preliminary construction of the student development model

The researcher’s initial model indicators of student development in undergraduate vocational education were based on China’s actual talent demand, the literature, and vocational education policies. Six experts (from the vocational education department, undergraduate vocational universities, career development planning, corporate human resources, technical executives, and student development studies) who were sufficiently familiar with the topic of this study and had worked in the field for ten years or more, were invited to participate in the indicator construction process. They discussed indicators and constructed a preliminary five-level student development model with the two elements of cognition and non-cognition, the four dimensions of knowledge, ability, value, and quality, eight factors, and 33 program indicators (Table 2).

**Table 2:** Preliminary construction of student development model indicators.

Element (2^nd^-level indicator)	Dimension (3^rd^-level indicator)	Factor (4^th^-level indicator)	Program (5^th^-level indicator)	Song KH et al.(2012) [22]	Ma Y(2015) [23]	Guo GJ et al.(2017) [24]	Li YZet al. (2019) [25]	Zhao ZQ (2019) [26]	Liu L (2016) [27]	Fang ZQ (2019) [28]	Chen Q (2020) [29]	Wen BY et al.(2020) [30]	Hu YH (2020) [31]	Wang J et al.(2020) [32]	Bowen (1977) [6]	Astin (1997) [33]	Chickering et al.(1993) [34]	Zhao XY(2013) [35]	Guo LB(2014) [36]	Wang WJ(2014) [37]	He XL(2019) [8]	Zhuo N(2019) [38]	Yang LJ et al.(2018) [39]
Cognition (B1)	Knowledge (C1)	General knowledge (D1)	Extensive coverage of relevant fields of knowledge (E1)	√	√		√	√						√		√			√	√	√		√
			Learn about science (E2)	√	√	√	√	√															
			Learn about the humanities (E3)		√	√	√		√				√	√									
			Learn about art (E4)		√	√												√					
		Professional knowledge (D2)	Professional basic knowledge (E5)	√		√	√	√	√	√		√		√	√			√		√			
			Deep professional theoretical knowledge (E6)	√			√		√	√	√		√	√					√		√		√
			Professional technical application knowledge (E7)			√							√										
	Ability (C2)	General ability (D3)	Good oral presentation ability (E8)	√	√	√												√	√		√	√	√
			Good writing ability (E9)	√	√	√												√	√	√	√	√	√
			Foreign language ability (E10)	√		√							√										
			Proficiency in information technology applications (E11)	√		√	√						√	√				√	√	√	√	√	√
			Organizational leadership ability (E12)	√		√							√						√	√	√		√
			Effective cooperation ability (E13)	√		√	√		√				√	√	√	√	√	√	√	√	√	√	√
			Self-learning ability (E14)		√	√			√				√	√			√	√	√	√			
		Professional ability (D4)	Job adaptability (E15)	√	√				√	√					√								
			Post operation ability (E16)	√		√		√	√	√	√	√		√			√						
			Ability to solve post problems (E17)	√	√	√	√		√				√					√	√	√	√		√
			Post innovation ability (E18)	√	√	√		√	√				√	√				√		√			
		Career development ability (D5)	Career planning ability (E19)										√						√		√	√	√
			Career transfer ability (E20)	√	√		√	√	√	√			√	√									
			Career conversion ability (E21)	√	√		√			√	√												
			Career improvement ability (E22)	√									√	√									
Non-cognition (B2)	Value (C3)	Value (D6)	Establishment of value (E23)	√		√		√						√	√	√		√	√				
			Personal outlook on the world and life (E24)	√		√		√											√		√		√
			Understanding of the culture and values of different groups (E25)																√	√	√		√
	Quality (C4)	Personal quality (D7)	Self-awareness (E26)	√		√							√	√		√		√	√				
			Personal character (E27)	√			√		√					√	√	√				√			
			Physical and mental health (E28)	√		√	√	√	√					√		√	√			√		√	
			Sense of responsibility (E29)	√	√		√		√				√	√			√	√		√		√	
		Professional quality (D8)	Professional ethics (E30)		√	√		√						√									
			Competitive awareness (E31)			√																√	
			Reverse thinking (E32)			√											√			√			
			Craftsman spirit (E33)	√					√				√	√									

### 3.2 Stage 2: Refining of student development model

This study conducted a first round of the expert survey to refine indicators and improve their reliability and comprehensiveness before screening the indicators. The first author distributed the indicator refinement questionnaire to 22 experts (the same experts as those surveyed for the FDM and AHP and not duplicated by the six experts) from January 4 to 28, 2022, by whom 20 surveys were returned. They provided their opinions on the modifying, adding, and merging indicators, explaining the reasons (Table 3). Then, the expert panel of six experts was invited to discuss further and analyze the experts’ advice to refine the student development indicators. The results are shown in Table 4.

**Table 3:** Results of the first round of the expert survey.

**Indicators to be refined**			
**Original No.**	**Indicator**	**Expert Suggestion**	**Handling Opinion**
C3	value	Value is part of quality. It is recommended to merge value and quality.	Merge C3 with C4.
C4	quality	Quality covers value. It is proposed to merge the two.	Merge C4 with C3.
E6	deep professional theoretical knowledge	Undergraduate vocational education emphasizes technical theory. Modify it to deep professional theoretical knowledge.	Modify E6.
E12	organizational leadership ability	Students work in technical skills, and enterprises do not demand high organizational leadership. So, modify it to some organizational leadership.	Modify E12.
**Indicators to be added**			
**Original No.**	**Indicators**	**Reasons for adding indicators**	**Handling Opinion**
E19	emergency handling ability	Students on the production line must possess excellent emergency handling ability to reduce accident damage.	The vocational ability module should include emergency handling ability.
E30	dialectical thinking	Students need to think dialectically and view technical skills jobs with a developmental eye.	The personal quality module should add dialectical thinking.
E34	legal awareness	Students must have rigorous legal awareness throughout their careers.	The vocational quality module needs to add legal awareness.

**Table 4:** Indicators of the refined student development model.

Goal (1^st^-level indicator)	Element (2^nd^-level indicator)	Dimension (3^rd^-level indicator)	Factor (4^th^-level indicator)	Program (5^th^-level indicator)
Student development (A)	Cognition (B1)	Knowledge (C1)	General knowledge (D1)	Extensive coverage of relevant fields of knowledge (E1)
				Learn about science (E2)
				Learn about the humanities (E3)
				Learn about art (E4)
			Professional knowledge (D2)	Professional basic knowledge (E5)
				Deep professional theoretical knowledge (E6)
				Professional technical application knowledge (E7)
		Ability (C2)	General ability (D3)	Good oral presentation ability (E8)
				Good writing ability (E9)
				Foreign language ability (E10)
				Proficiency in information technology applications (E11)
				Some organizational leadership ability (E12)
				Effective cooperation ability (E13)
				Self-learning ability (E14)
			Professional ability (D4)	Job adaptability (E15)
				Post operation ability (E16)
				Ability to solve post problems (E17)
				Post innovation ability (E18)
				Emergency handling ability (E19)
			Career development ability (D5)	Career planning ability (E20)
				Career transfer ability (E21)
				Career conversion ability (E22)
				Career improvement ability (E23)
	Non-cognition (B2)	Quality (C3)	Value (D6)	Establishment of value (E24)
				Personal outlook on the world and life (E25)
				Understanding of the culture and values of different groups (E26)
			Personal quality (D7)	Self-awareness (E27)
				Personal character (E28)
				Physical and mental health (E29)
				Sense of responsibility (E30)
				Dialectical thinking (E31)
			Professional quality (D8)	Professional ethics (E32)
				Competitive awareness (E33)
				Reverse thinking (E34)
				Craftsman spirit (E35)
				Legal awareness (E36)

### 3.3 Stage 3: Screening indicators using the FDM

The first author conducted a second round of expert surveys between February 15 and 29, 2022, using the FDM to screen the student development model’s key indicators. The Delphi method was first proposed by Dalkey and Helmer in 1963 to obtain group decisions by consulting experts [40]. However, in many situations, the judgment of experts cannot be quantified precisely. Murray, Pipino, and Gigch proposed the Fuzzy Delphi method (FDM) in 1985 to overcome the limitations of the traditional Delphi method using fuzzy theory. Compared with the conventional Delphi method, the FDM has the following advantages: 1. Fewer surveys; 2. Each expert’s opinions are considered and integrated; 3. Fuzzy theory considers the expert’s uncertain and subjective opinions; 4. Reduced time and cost required for surveys [41].

FDM screening indicators generally include the following steps: establishing the model hierarchy and designing the expert questionnaire, collecting experts’ decision-making opinions, checking the degree of consensus, and applying the threshold value to screen the indicators [42]. This study analyzed experts’ opinions involving the double-triangular fuzzy number [20] and checked for a consensus using the gray area [43]. The four steps of the FDM to screen indicators in this study are:

Step 1: Establish a hierarchy and design a questionnaire.

The five-level hierarchy of the model indicators is shown in Table 4. The expert survey consists of two values: (1) the indicator’s “single value” indicates the quantitative value of its importance, and (2) the interval value for each indicator. The minimum of this interval value indicates the expert’s “conservatively perceived value (C)” for the quantitative score of the indicator, and the maximum means the expert’s “optimistically perceived value (O)” for it. The above values are all different integers ranging from 1 to 9, with larger values indicating greater importance [44].

Step 2: Establish double triangular fuzzy numbers.

**Fig 2:**
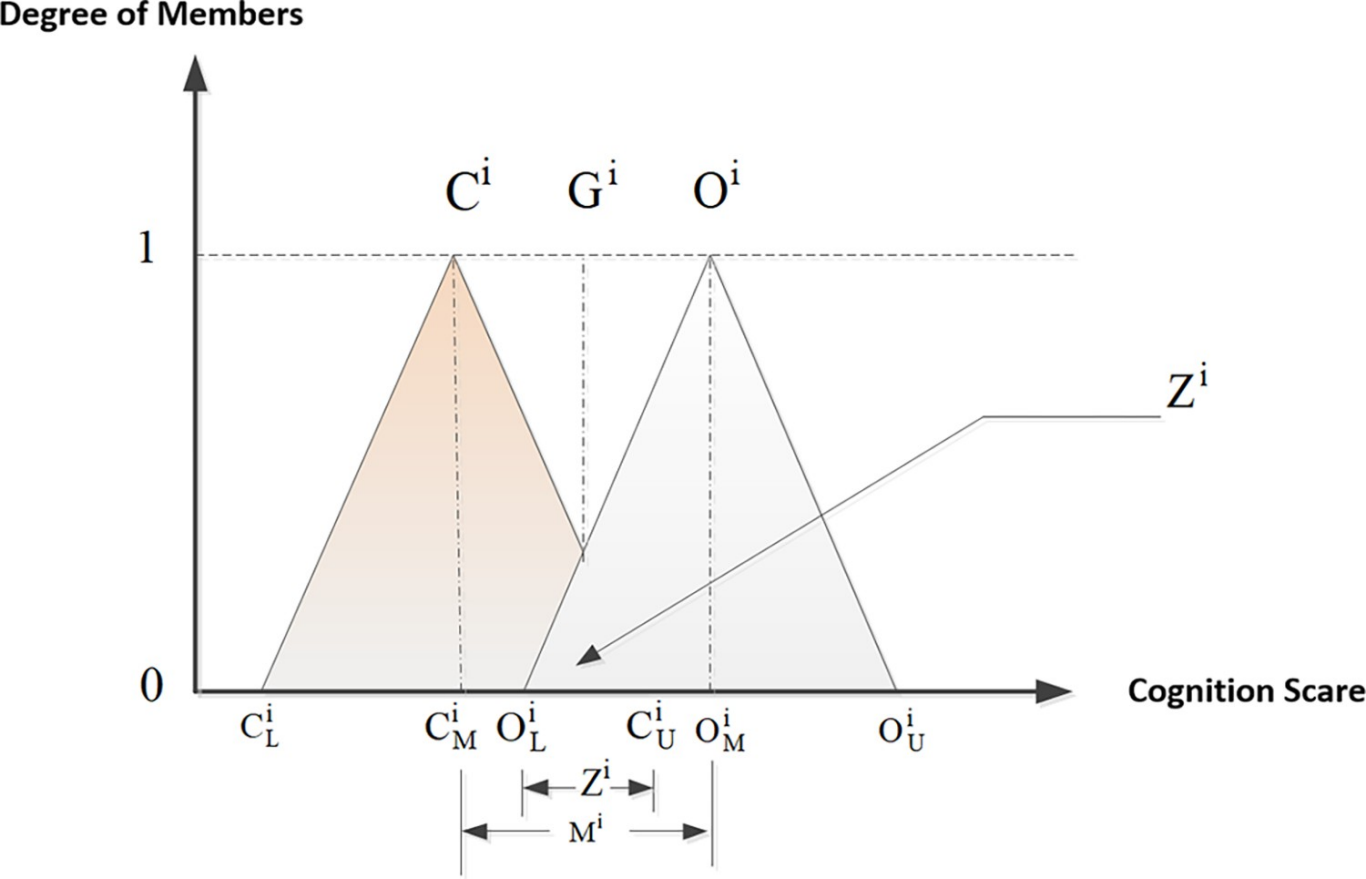
Double triangular fuzzy numbers [46].

The minimum $D_{L}^{i}$, maximum $D_{U}^{i}$ and geometric mean $D_{M}^{i}$ of the “single value,” the minimum $C_{L}^{i}$, maximum $C_{U}^{i}$ and geometric mean $C_{M}^{i}$ of the “conservatively perceived value” and the minimum $O_{L}^{i}$, maximum $O_{U}^{i}$ and geometric mean $O_{M}^{i}$ of the “optimistically perceived value” given by each expert for each indicator i are calculated, and the corresponding triangular fuzzy number of “conservatively perceived value” $C^{i}=(C_{L}^{i}+C_{M}^{i}+C_{U}^{i})$ and that of the “optimistically perceived value” $O^{i}=(O_{L}^{i}+O_{M}^{i}+O_{U}^{i})$ for each indicator i are established [45], as shown in Fig 2.

Step 3: Check the degree of consensus reached by experts.

This step is to check how the two triangular fuzzy numbers overlap to determine if the experts can reach a consensus [43].

- Check if there is a gray zone. When $C_{U}^{i}\leq C_{L}^{i}$, it means that the two triangular fuzzy numbers do not overlap, and no gray zone exists, which indicates that experts’ opinions do not converge. Then conduct the next round of surveys, attaching the statistics of this round. When $C_{U}^{i}>C_{L}^{i}$, it indicates that they overlap and a gray area exists. Then calculate the check value for the gray zone to verify whether the experts reach a consensus.
- Calculate the check value for the gray zone. To assess if the experts’ opinions have converged, the fuzzy gray zone $Z^{i}=C_{U}^{i}-O_{L}^{i}$ is compared to the range $M^{i}=O_{M}^{i}-C_{M}^{i}$ of the geometric mean of the “conservatively perceived value” and “optimistically perceived value.” If *M*^*i*^−*Z*^*i*^ > 0, it means that the experts reached a consensus. If *M*^*i*^−*Z*^*i*^ < 0, it indicates there is a gray area with no expert consensus. Therefore, it is necessary to consult the statistics on the “conservatively perceived value” and “optimistically perceived value” of indicators that do not converge to experts for reference, and another survey has to be carried out, repeating steps 1 to 3 until all the indicators reach consensus.

Step 4: Calculate the threshold value of the screening indicators.

When the importance of each indicator reaches convergence, the geometric mean of the “single value” and the “conservatively and optimistically perceived value” for each indicator is geometrically averaged to obtain its consensus value, *G*^*i*^. The larger a *G*^*i*^, the higher the consensus and importance of the indicator. The geometric mean of the “single value,” the “conservatively perceived value,” and the “optimistically perceived value” for all indicators is then geometrically averaged to determine the indicator screening threshold *T*^*i*^. When the *G*^*i*^<*T*^*i*^, the indicator is deleted. If not, it is retained.

### 3.4 Stage 4: Determining weight by applying the AHP

The weight reflects an indicator’s importance in the mode, and its reasonableness directly affects the scientific robustness of student development assessment. The first author used the Analytical Hierarchy Process (AHP) to assign indicator weight in a third round of expert surveys from April 6 to 27, 2022. Saaty proposed the AHP in the mid-1970s, which is a multi-objective decision-making method that combines qualitative and quantitative analyses [47]. The Saaty scale is adopted, and the pairwise comparisons [48] are used to assign the student development weights. The AHP involves four steps:

Step 1: Establish a hierarchy.

The hierarchy structure divides a complex problem into indicators grouped by attributes to form different levels. The upper-level indicators govern the same-level indicators and guide some of the lower-level indicators. The structure divides decision-making objectives, factors, and objects into high, middle, and low levels based on their relationships.

Step 2: Create the pairwise comparison matrix.

The weight questionnaire is designed based on the indicator hierarchy. Experts make pairwise comparisons of same-level indicators, and the rating scale uses Saaty’s relative importance scale ranging from 1 to 9 [49]. The higher the number, the greater the importance of one indicator relative to another. The comparison results are organized to form a pairwise comparison matrix to obtain the eigenvectors and eigenvalues.

Step 3: Carry out a consistency test.

The judgment of expert consistency is based on the consistency ratio (CR), calculated by dividing the consistency indicator for the judgment matrix by the corresponding random matrix (RI). Saaty (1980) suggested that CI≤0.1 indicates that the pairwise comparison matrix is consistent. When CI>0.1, it indicates inconsistency. When CR<0.1, it indicates that its consistency is satisfactory. If CR≥0.1, the consistency test is not passed, and another round of expert surveys is carried out until it is consistent. The test formula is as follows:

$$\mathrm{CI}=\frac{\lambda_{\max}‐n}{n-1}$$ (1)

CR=CI/RI (2)

Step4: Calculate indicator weight.

The eigenvector method is used to calculate the maximum eigenroot vector of each pairwise comparison matrix, which is normalized to obtain the weight of each indicator. Then, the local weight is calculated by averaging each indicator weight of all experts.

### 3.5 Selection of survey experts

The survey experts had to be familiar with and cooperate with this study. Zhong Zhengwei et al. believe a group of experts consisting of more than ten but less than 30 members makes the fewest group attribution errors and establishes the highest credibility [41]. Twenty-two experts surveyed in this study met the following two criteria.

Criterion 1: Closely related fields of this study. Selecting 22 experts based on our research characteristics requires both authoritative academic experts in the field and experts from the front line, in this case, including two student development research experts, six undergraduate vocational education talent cultivation research experts, two experts in career planning for college students, two vocational education policy development experts, four enterprise human resources experts, and four technical experts. To be chosen as an expert in this study, individuals had to have at least an associate senior title and ten years or more of experience in the field. Specific information on the experts in each area is shown in Table 5.

**Table 5:** Statistical analysis table of basic data of survey experts.

Type	Title or position	Number	Service length	Specification
Management consultants in human resources	Human resources manager	4	≥10years	1. Familiar with the company’s talent needs and job matching.
				2. Conduct over more than 100 recruitment interviews.
Undergraduate vocational education experts	Professor or associate professor	6	≥10years	1. Set university talent training objectives.
				2. Published over 5 vocational education papers.
Career planning experts	Professor or associate professor	2	≥10years	1. Familiar with the undergraduate vocational education student development.
				2. Supervise the vocational education student development for 10 years or more.
				3. Receive honors in vocational education at or above the municipal level.
Vocational education sector experts	Education sector head	2	≥10years	1. Formulate and implement vocational education policies for 10 years.
				2. Familiar with undergraduate vocational education talent cultivation objectives.
				3. Receive honors in vocational education at or above the municipal level.
Technologist	Senior or associate senior engineer	4	≥10years	1. Familiar with the competence structure of undergraduate vocational education talents.
				2. Have worked in an applied talent position for 10 years or more.
Student development research specialist	Professor or associate professor	4	≥10years	1. Familiarity with the student development model, dimension, etc.
				2. Published over 5 student development papers.

Criterion 2: Expert authority coefficient (*C*_*r*_). The expert authority coefficient (*C*_*r*_) is the arithmetic mean of the judgment coefficient (*C*_*a*_) and familiarity coefficient (*C*_*s*_), namely, $C_{r}=\left(C_{a}+C_{s}\right)/2,\;C_{r}\geq 0.70$ indicates acceptable reliability. *C*_*a*_ represents the expert judgment basis [50]. In this study, experts used terms such as “practical experience,” “logical reasoning,” “domestic and international knowledge,” and “intuition” as judgment criteria (Table 6). *C*_*s*_ represents an expert’s familiarity with the problem, and its value ranges from 0 to 1 (1 = very familiar, 0.75 = familiar, 0.5 = relatively familiar, 0.25 = generally familiar, 0 = not very familiar). The closer *C*_*r*_ is to 1, the greater an expert’s authority and the higher the reliability of the survey results.

**Table 6:** Judgment basis and the degree of influence.

Judgment basis	Influence degree (*C*_*a*_)		
	High Impact	Moderate Impact	Low Impact
Practical experience	0.5	0.4	0.3
Logical reasoning	0.3	0.2	0.1
Domestic and international knowledge	0.1	0.1	0.1
Intuition	0.1	0.1	0.1
Total	1.0	0.8	0.6

## 4. Data analysis and results

### 4.1 Analyzing experts’ authority coefficients

The authority coefficients of 20 experts who responded positively in the first round are between 0.7 and 1.0, with a mean of 0.91, which indicates that these experts are authoritative and that the survey results are reliable. The second and third rounds of expert surveys only surveyed the 20 experts who had responded positively in the first round.

### 4.2 FDM analysis results

The expert panel decided to conduct a second round of expert surveys for the 5^th^-level indicators since the 2^nd^- to 4^th^-leve indicators had a high degree of expert consensus and a well-established theoretical basis. The survey was carried out according to the operational steps of the FDM, and Microsoft Excel was used to analyze the survey data. The results are shown in Table 7. All of the indicators’ gray zone test values *Z*^*i*^ are greater than 0, indicating the presence of gray areas. Their *M*^*i*^−*Z*^*i*^ > 0 means that experts’ opinions tend to be consistent, and the indicator is convergent.

**Table 7:** Results of the screening indicator.

Indicator No.	*Z* ^*i*^	*M* ^*i*^	*M*^*i*^−*Z*^*i*^	*G* ^*i*^	Del or not	Indicator No.	*Z* ^*i*^	*M* ^*i*^	*M*^*i*^−*Z*^*i*^	*G* ^*i*^	Del or not
E1	1	1.60	0.60	2.12	Del	E19	1	2.15	1.15	7.97	
E2	1	2.10	1.10	7.70		E20	1	2.20	1.20	7.73	
E3	1	2.10	1.10	7.68		E21	1	2.20	1.20	7.72	
E4	1	2.50	1.50	7.67		E22	1	2.00	1.00	8.02	
E5	1	2.05	1.05	7.65		E23	1	1.95	0.95	7.87	
E6	1	2.25	1.25	7.93		E24	1	1.85	0.85	8.28	
E7	1	2.25	1.25	7.93		E25	1	2.05	1.05	8.15	
E8	1	2.20	1.20	7.95		E26	1	2.15	1.15	7.70	
E9	1	2.00	1.00	7.70		E27	1	2.05	1.05	8.07	
E10	1	1.85	0.85	7.72		E28	1	1.95	0.95	8.20	
E11	1	2.25	1.25	7.82		E29	1	2.05	1.05	8.20	
E12	1	2.05	1.05	7.70		E30	1	2.00	1.00	8.23	
E13	2	2.05	0.05	7.92		E31	1	2.50	1.50	7.68	
E14	1	2.15	1.15	8.05		E32	1	2.10	1.10	8.18	
E15	1	2.20	1.20	7.95		E33	2	2.10	0.10	3.90	Del
E16	1	2.00	1.00	8.03		E34	4	5.90	1.90	4.23	Del
E17	1	2.05	1.05	8.15		E35	1	2.60	1.60	7.70	
E18	1	2.10	1.10	7.83		E36	1	2.20	1.20	8.03	

The expert consensus value *G*^*i*^ is an essential factor in screening indicators. The higher it is, the greater its importance. As for the screening indicators, they are determined by the decision threshold *T*^*i*^. The threshold will directly affect the number of screening indicators. Yao Kaichao (2022) believed that the threshold could be set to 6, excluding indicators with G^i^ below 6 [46]. Chen Wenliang (2021) took the arithmetic mean of all indicators *G*^*i*^ as the threshold and deleted indicators with a *G*^*i*^ value below the mean [51]. This study used the arithmetic mean of all indicators, 7.54, as the decision threshold (above 6) to reduce human intervention. As shown in Table 7, E1, E33, and E34 were deleted since their *G*^*i*^ values were below 7.54. The other 33 indicators were retained (Table 8), with a consensus value of 7.6 or higher, whereas the three deleted indicators had a value of 4.5 or less. The results indicates that academics, industry, and government departments have reached a high degree of consensus on the student development indicators, thus providing a convincing basis for constructing a student development model.

**Table 8:** Results of screening student development indicators.

Goal (1^st^-level indicator)	Element (2^nd^-level indicator)	Dimension (3^rd^-level indicator)	Factor (4^th^-level indicator)	Program (5^th^-level indicator)
Student development (A)	Cognition (B1)	Knowledge (C1)	General knowledge (D1)	Learn about science (E1)
				Learn about the humanities (E2)
				Learn about art (E3)
			Professional knowledge (D2)	Professional basic knowledge (E4)
				Deep professional theoretical knowledge (E5)
				Professional technical application knowledge (E6)
		Ability (C2)	General ability (D3)	Good oral presentation ability (E7)
				Good writing ability (E8)
				Foreign language ability (E9)
				Proficiency in information technology applications (E10)
				Some organizational leadership ability (E11)
				Effective cooperation ability (E12)
				Self-learning ability (E13)
			Professional ability (D4)	Job adaptability (E14)
				Post operation ability (E15)
				Ability to solve post problems (E16)
				Post innovation ability (E17)
				Emergency handling ability (E18)
			Career development ability (D5)	Career planning ability (E19)
				Career transfer ability (E20)
				Career conversion ability (E21)
				Career improvement ability (E22)
	Non-cognition (B2)	Quality (C3)	Value (D6)	Establishment of value (E23)
				Personal outlook on the world and life (E24)
				Understanding of the culture and values of different groups (E25)
			Personal quality (D7)	Self-awareness (E26)
				Personal character (E27)
				Physical and mental health (E28)
				Sense of responsibility (E29)
				Dialectical thinking (E30)
			Professional quality (D8)	Professional ethics (E31)
				Craftsman spirit (E32)
				Legal awareness (E33)

### 4.3 AHP analysis results

This study established a five-level student development model (Table 8). Researchers believe that assigning weights to too many levels and indicators makes pairwise comparisons complicated and laborious, requiring considerable patience by experts. Researchers, therefore, argued that methods or procedures should be optimized or simplified [46]. The expert panel made each 5^th^-level indicator equally crucial to the 4^th^-level indicator. Therefore, this study surveyed the weights of 2^nd^- to 4^th^-level indicators.

Pairwise comparison matrices were created based on the third round of expert survey data. They all passed the consistency test because they met the condition of CR<0.1. The pairwise comparison matrices created by Expert 1 are shown in Table 9.

**Table 9:** Pairwise comparison matrices of A, B1, C1, C2, and C3.

Indicator	Pairwise comparison matrix					
1^st^-level indicator A	A	B1		B2		*W*_*i*_
	B1	1		1/2		0.33
	B2	2		1		0.67
	CR = 0.00, the maximum feature value of the weight vector *λ* = 2.00					
2^nd^-level indicator B1	B1	C1		C2		*W*_*i*_
	C1	1		2		0.67
	C2	1/2		1		0.33
	CR = 0.00, the maximum feature value of the weight vector *λ* = 2.00					
3^rd^- level indicator C1	C1	D1		D2		*W*_*i*_
	D1	1		1/2		0.33
	D2	2		1		0.67
	CR = 0.00, the maximum feature value of the weight vector *λ* =2.00					
3^rd^-level indicator C2	C2	D3	D4		D5	*W*_*i*_
	D3	1	1/3		1/5	0.11
	D4	3	1		1/2	0.31
	D5	5	2		1	0.58
	CR = 0.00, the maximum feature value of the weight vector *λ* = 3.00					
3^rd^- level indicator C3	C3	D6	D7		D8	*W*_*i*_
	D6	1	2		2	0.50
	D7	1/2	1		1	0.25
	D8	1/2	1		1	0.25
	CR = 0.00, the maximum feature value of the weight vector *λ* = 3.00					

Local weights, or relative weights, were obtained based on the pairwise comparison matrices. It is each indicator’s importance relative to its upper-level indicator. Based on the considerations of easy judgment and simple calculation, this study used the arithmetic mean of experts’ weights to obtain the local weight of the indicator and then calculated the global weight, also referred to as the absolute weight, which is the indicator’s weight on the same level. The results are shown in Table 10. The five-point Likert scale score was assigned to each 5^th^-level indicator (particularly much improvement = 5, a lot of improvement = 4, general improvement = 3, slight improvement = 2, almost no improvement = 1) to develop the student development assessment instrument.

**Table 10:** Final indicator weight and ranking.

1^st^-level indicator	2^nd^-level indicator	Weight	3^rd^-level indicator	Relative weight	Absolute weight	Ranking	4^th^-level indicator	Relative weight	Absolute weight	Ranking
Student development (A)	Cognition (B1)	0.50	Knowledge (C1)	0.33	0.17	3	General knowledge (D1)	0.37	0.06	7
							Professional knowledge (D2)	0.63	0.10	6
			Ability (C2)	0.67	0.33	2	General ability (D3)	0.11	0.04	8
							Professional ability (D4)	0.32	0.11	5
							Career development ability (D5)	0.57	0.19	2
	Non-cognition (B2)	0.50	Quality (C3)	1	0.50	1	Value (D6)	0.47	0.24	1
							Personal quality (D7)	0.28	0.14	3
							Professional quality (D8)	0.25	0.16	4

The results of this study showed that the two 2^nd^-level indicators, cognitive and non-cognitive, are equally significant to student development. The three 3^rd^-level indicators are ranked in importance in terms of quality, ability, and knowledge development, indicating that experts pay more attention to students’ quality and ability development. In cognitive development, ability development is significantly more important than knowledge development. This result is consistent with socialist education with Chinese characteristics, prioritizing moral education, ability, and comprehensive development. Among the eight 4^th^-level indicators, the top five are value (D6), career development ability (D5), personal quality (D7), vocational quality (D8), and vocational ability (D4), which indicates that society places the greatest emphasis on the students’ value development, followed by the ability and quality requirements associated with the characteristics of undergraduate vocational education. The indicator importance ranking is generally consistent with that of the FDM screening indicators, thus verifying the validity of this study’s student development model.

## 5. Discussion and recommendations

Based on survey data from 20 experts in six fields, this study used the FDM and AHP to design a reliable student development model and assessment instrument that fills research gaps. The proposed model is comprehensive, pertinent, systematic, and scientific, as shown by its analysis.

Regarding comprehensiveness, the model depicted student development elements from five levels, three dimensions, and eight factors, clarified its constituents and ability observation points, and comprehensively and fully reflected the essential attributes of student development in undergraduate vocational education. In addition, the researcher and experts constructed the student development model from the perspective of the social and economic development requirements for high-quality technical and skilled talent and individual student development. This study followed the student development theory and divided it into cognitive and non-cognitive development, which have been well-respected among researchers. Other researchers focused more on students’ learning engagement or the influence of various external factors on student development [8, 35, 36]. Their indicators are more macroscopic and fewer in number, while the model presented in this study is rich in levels and comprehensive in indicators.

Pertinence is mainly manifested in the following ways. First, other researchers’ student development models include basic knowledge and abilities indicators that are somewhat universal [4, 8, 35, 36]. Comparatively, this study focused on the outstanding vocational and practical characteristics of undergraduate vocational education and used vocational quality, vocational ability, and career development ability as the 4^th^-level indicators. The 5^th^-level indicators included emergency handling ability, post-operation and problem-solving ability, transfer ability, conversion ability, and craftsmanship, which are essential abilities and qualities for students in the production line job groups. However, student development indicators of vocational colleges emphasize specific techniques and skills needed in a certain position or craftsmanship, and require very little theoretical knowledge, innovation, and developmental abilities. Indicators of general undergraduate universities focus on systematic and complete theoretical knowledge, strong dialectical thinking, and weak practical abilities. Student development indicators and their weights will vary across the different categories of universities due to the above differences. Second, it is worth noting that the model presented in our study is localized to be applied in evaluating student development in China and may not be appropriate for evaluating student development in other countries. Chinese culture is an ethical culture that places human development within the context of interpersonal relationships and values human virtues and cooperation. In comparison, Western culture emphasizes individual growth and is an individualist culture [52]. Such cultural differences inevitably lead to differences in student development indicators in different countries, and even the same indicator may be weighted inconsistently across countries.

For example, undergraduate vocational education in Germany and the United States starts earlier, so students already possessed post-practice and problem-solving abilities. They now emphasize cultivating students’ innovative spirit and creative ability [53]. However, China’s undergraduate vocational education has just begun emphasizing students’ basic vocational and career development abilities. Third, experts’ weights are consistent with China’s educational standard of “morality, ability, and knowledge” [54], prioritizing national conditions and fully embodying socialist universities with Chinese characteristics.

As far as systematicity is concerned, it is reflected in the indicator design, which considers the connections between indicators and levels. Each indicator performs its functions and roles and is intertwined with others to constitute an organic whole. Previous research only divided model dimensions but neglected their level of performance. This study examined the model indicators holistically, focusing on the systematic composition of student development and subdividing them into levels to show the entire pattern of student development in an all-round and multi-level way. The division into levels for each dimension corresponds to the student development stage and is well differentiated. For instance, ability development is divided into general, vocational, and career development ability in ascending order of difficulty, and quality development is subdivided into value, personal quality, and vocational quality from high to low, according to importance. The indicators were assigned weights according to their importance, and the model indicators were designed from a global perspective. The application of the FDM and AHP standardized the screening process and the assignment of weights to the indicators and allowed for the construction of the student development model indicators with systematic characteristics.

From the scientific perspective, the authority and consensus of the experts included in our study are high, and the results are reliable. This is also reflected in the indicator selection and design method. Regarding indicator selection, this study added two steps based on the literature before conducting the Delphi expert survey. First, the expert panel, consisting of six experts, was invited to discuss and analyze the indicators. Second, the preliminary indicators were determined after the indicators were refined through the expert survey, which included 20 experts, so repeated discussions and surveys improved the scientific rigor of the indicators. In terms of the indicator design method, the FDM overcame human subjective ambiguity in indicator analysis. The AHP was used to determine indicator weights by considering the model’s hierarchical nature and the interrelationships between levels. These two methods reduced indicator selection subjectivity and arbitrariness while also avoiding the problems of the simplification and superficiality of indicators caused by the singular use of qualitative or quantitative methods. The experts unanimously agreed that this study’s model and assessment instrument could be widely used in undergraduate vocational university student development.

Despite its contributions, this study also has some limitations. First, the experts have a high degree of authority and consistency in data analysis, and the designed indicators have a certain scientific nature. Nevertheless, our study is part of ongoing academic research, and the constructed assessment index system has not yet been empirically verified. Second, the subjects of this study are experts from different fields, and the number of experts per field was uneven. Their positions and opinions may also differ, although they all belonged to the decision-making group.

## 6. Conclusion

This student development model constructed in this study is China’s first set of indicators of an “undergraduate vocational university student development model.” Before this study, evidence of undergraduate vocational education student development was merely anecdotal. The academic rigor and transparency of our study process make a novel contribution to the vocational education and student development literature and thus can help other countries consider ways to transfer the findings to their context. Furthermore, our study’s five-level model is essential and actionable for undergraduate vocational education student development practice in China. Education authorities can use the model to assess the quality of talent cultivation at undergraduate vocational universities. Universities can use this instrument to assess student development, diagnose related problems, and collect empirical data to improve it. They can also optimize the allocation of educational and teaching resources based on the indicator weights to quickly cultivate the urgently needed talent and promote the high-quality development of China’s economy and society. It is expected that the government, authorities, and universities will make a coordinated effort to pilot the model in some universities, further improve it based on practice, and then establish a national standard for all undergraduate vocational universities as soon as possible.

This study marks the beginning of undergraduate vocational university student development research, and there is still a considerable need for further research. First, future researchers can use our proposed model to track student development data and assess student groups’ development levels and characteristics in different grades, disciplines, and universities to provide empirical data for optimizing the model and improving student development. Second, this study was carried out by Chinese experts and undergraduate vocational universities, and future research can apply the proposed research methods to other countries and universities by conducting similar surveys and comparing the survey results to learn from each other. Third, researchers can promote students’ outstanding development in terms of the critical indicators by conducting in-depth studies on the specific implementation of each key indicator. Fourth, in future research, the indicators can be further revised according to the actual situation of the application of the indicators to maximize their adaptation to actual needs and achieve the highest possible objectivity and accuracy. Fifth, future research can consider the number of survey experts in different fields and weigh their relative importance to make the survey results more scientific.

## Supporting information

S1 Dataset Data used in this study. S1A, S1D and S1G are the first, second and third rounds of expert questionnaires respectively. S1C, S1E and S1H are the raw data of the first, second and third rounds of expert surveys respectively. S1B is the authoritative level data for the first round of expert surveys. S1F is the statistical analysis data of screening indicators in the second round of expert surveys. S1I is the statistical analysis data of indicator weight in the third round of expert surveys.
(ZIP)
