# Supplementary material for: Constructing a student development model for undergraduate vocational universities in China using the Fuzzy Delphi Method and Analytic Hierarchy Process
Source: PLoS One. 2024 Mar 22;19(3):e0301017. doi: 10.1371/journal.pone.0301017 (PMC10959347; doi:10.1371/journal.pone.0301017)
Supplement: S1 Dataset — S1A, S1D and S1G are the first, second and third rounds of expert questionnaires respectively. S1C, S1E and S1H are the raw data of the first, second and third rounds of expert surveys respectively. S1B is the authoritative level data for the first round of expert surveys. S1F is the statistical analysis data of screening indicators in the second round of expert surveys. S1I is the statistical analysis data of indicator weight in the third round of expert surveys. (ZIP) [file pone.0301017.s001.zip › S1 Dataset/S1A Dataset (First round of expert survey questionnaire).docx]

**Expert Consultation Questionnaire on Student Development Construct Indexes in Undergraduate Vocational Universities-Index Refinement**

Dear experts，

I am Qiaona Xing, a Ph.D. student in Philosophy (Social Science) at Universiti Tunku Abdul Rahman, Malaysia. I am researching “Applying Fuzzy Delphi Technique to design student development construct in undergraduate vocational universities”.

This thesis has a distinctive problem orientation. It focuses on developing vocational education in China in response to the shortage of high-quality tech talents in economic transformation and industrial upgrading in recent years. It is dedicated to “how to design the student development in undergraduate vocational universities” and “what core qualities and abilities should be cultivated in undergraduate vocational education”.

In this thesis, the undergraduate vocational university student developmental construct refers to the knowledge, ability, and quality that Chinese undergraduate vocational university students should possess after completing their university-level studies. It refers to what students have learned and what they can do when they graduate. This thesis aims to design a student development construct for undergraduate vocational universities, which refers to the specific knowledge, ability, and quality students must have by the time they graduate.

It is a theoretical guide for designing and implementing student development in undergraduate vocational universities. It will help them improve the talent training quality and cultivate talents aligned with China’s economic and social development.

This thesis initially drew up an undergraduate vocational university student developmental construct after a preliminary study (as shown in the table below). This consultation explores what knowledge, ability, and quality undergraduate vocational university students should possess upon graduation based on the goals and characteristics of talent training in undergraduate vocational education. Experts are invited to provide comments and suggestions on modifying, retaining, merging, and adding to the student development construct indexes listed in Table 1 to improve further the student development construct of undergraduate vocational universities designed in this thesis and provide scientific theoretical guidance and data support for undergraduate vocational universities to cultivate high-quality tech talents who meet the needs of social and economic development.

It is a great honor to invite you as the consulting expert for this thesis. Your comments or suggestions will significantly help the successful completion of this thesis. With your valuable opinions, I hope we can jointly discuss a scientific and reasonable undergraduate vocational education student development construct. Thank you for your help and support. I am very grateful!

Xing Qiaona, a Ph.D. candidate at UTAR University

April 2021

**Preliminary Designed Undergraduate Vocational Education Student Development Construct：**

| **Goal**  **(1^st^-level index)** | **Element**  **(2^nd^-level index)** | **Dimension**  **(3^rd^-level index)** | **Factor**  **(4^th^-level index)** | **Index**  **(5^th^-level index)** |
| --- | --- | --- | --- | --- |
| student development | cognitive development | knowledge development | general knowledge | extensive coverage of relevant fields of knowledge |
|  |  |  |  | learn about science |
|  |  |  |  | learn about the humanities |
|  |  |  |  | learn about art |
|  |  |  | professional knowledge | professional basic knowledge |
|  |  |  |  | deep professional theoretical knowledge |
|  |  |  |  | professional technical application knowledge |
|  |  | ability development | general ability | good oral presentation ability |
|  |  |  |  | well-written expression ability |
|  |  |  |  | foreign language application ability |
|  |  |  |  | proficiency in the application of information technology |
|  |  |  |  | organizational leadership ability |
|  |  |  |  | ability to cooperate effectively with others |
|  |  |  |  | self-learning ability |
|  |  |  | professional ability | job adaptability |
|  |  |  |  | post operation ability |
|  |  |  |  | ability to solve problems on the job |
|  |  |  |  | ability to innovate professional positions |
|  |  |  | career development ability | career planning ability |
|  |  |  |  | career changeability |
|  |  |  |  | career mobility ability |
|  |  |  |  | career advancement ability |
|  | non-  cognitive development | value  development | value | establishment of value |
|  |  |  |  | personal outlook on the world and life |
|  |  |  |  | understanding of the culture and values of different groups |
|  |  | quality  development | personal quality | self-awareness |
|  |  |  |  | personal character |
|  |  |  |  | physical and mental health |
|  |  |  |  | sense of responsibility |
|  |  |  | professional quality | professional ethics |
|  |  |  |  | competitive awareness |
|  |  |  |  | reverse thinking |
|  |  |  |  | craftsman spirit |

**Completion instructions：**

1. This thesis considers what knowledge, ability, and quality undergraduate vocational university students must have when they graduate. The consultation results on student development construct indexes for undergraduate vocational universities will be used to reverse the design of student development pathways and evaluation systems. The purpose of this consultation is to determine: the rationality and appropriateness of the developmental construct of students in undergraduate vocational universities.

2. Please fill in your familiarity with undergraduate vocational education students’ development construct indexes and your judgment based on the actual situation.

3. You are invited to review the preliminary construct indexes for student development in undergraduate vocational education designed in this thesis. If you have any comments on modifying, reserving, or combining the indexes, please indicate the category in the “Disposal comments” column and fill in the “Expert comments and suggestions” column. If you wish to add a new index, please enter the new number, the content of the index, and the reason for the addition in the “New index” field.

**Part I Background information**

1. Your title: □Professor □Associate Professor □Lecturer □Other__

2. Your position ( )

3. Number of years in your current position: □Less than 1 year □1-5 years □6-9 years □10-15 years □16-20 years □More than 20 years

4. Your age: □30-40 years old □40-50 years old □50 years old or above

5. Your degree: □ Specialist □ Bachelor □Postgraduate □Doctorate

**Part II Basis for judgment**

1. How familiar are you with the undergraduate vocational education student development construct indexes?

□Very familiar □Quite familiar □Generally familiar □Not very familiar □Very unfamiliar

2. The extent to which your judgment of the construct indexes of student development in undergraduate vocational education is influenced by the following factors.

Practical experience: □Highly influential □Generally influential □Generally familiar

Logical reasoning: □Highly influential □Generally influential □Generally familiar

Knowledge of national and international: □Highly influential □Fairly influential □Fairly familiar

Intuition: □High impact □Average impact □Average familiarity

**Part Ⅲ Expert consultation on student development construct indexes in undergraduate vocational universities**

1. The researcher believes that student development in undergraduate vocational universities consists of two 2^nd^-level indexes, cognitive and non-cognitive development.

| **Indexes that need refine** | | | | | |
| --- | --- | --- | --- | --- | --- |
| **No.** | **Index** | **Disposal comments**  **(modify, retain, merge)** | | | **Expert comments and suggestions** |
| 1 | cognitive development | □ Modify | □ Retain | □ Merge |  |
| 2 | non-cognitive development | □ Modify | □ Retain | □ Merge |  |
| **Indexes that need to be added** | | | | | |
| **Add number** | **Index** | **Reasons for increasing the index** | | | |
|  |  |  | | | |
|  |  |  | | | |
|  |  |  | | | |

2. According to the researcher, the construct indexes of student development in undergraduate vocational universities are divided into knowledge and ability development for cognitive development and value and quality development for non-cognitive development. Do you think this classification or expression is reasonable? Please fill in the table below with your comments on modifications, retaining, mergers, and additions.

| **Indexes that need perfect** | | | | | |
| --- | --- | --- | --- | --- | --- |
| **No.** | **Index** | **Disposal comments**  **(modify, retain, merge)** | | | **Expert comments and suggestions** |
| 1-1 | knowledge development | □ Modify | □ Retain | □ Merge |  |
| 1-2 | ability development | □ Modify | □ Retain | □ Merge |  |
| 2-1 | value development | □ Modify | □ Retain | □ Merge |  |
| 2-2 | quality development | □ Modify | □ Retain | □ Merge |  |
| **Indexes that need to be added** | | | | | |
| **Add number** | **Index** | **Reasons for increasing the index** | | | |
|  |  |  | | | |
|  |  |  | | | |
|  |  |  | | | |

3. The researcher believes that the four dimensions of knowledge, ability, value, and quality of undergraduate vocational university student development can be further refined. Knowledge is categorized as general and professional knowledge; ability is divided into general, professional, and career development ability; and qualify is categorized as personal and professional. If you think this classification or expression is unreasonable, please fill in the table below with your comments on modifications, retains, mergers, and additions.

| **Indexes that need perfect** | | | | | |
| --- | --- | --- | --- | --- | --- |
| **No.** | **Index** | **Disposal comments**  **(modify, retain, merge)** | | | **Expert comments and suggestions** |
| 1-1-1 | extensive coverage of relevant fields of knowledge | □ Modify | □ Retain | □ Merge |  |
| 1-1-2 | learn about science | □ Modify | □ Retain | □ Merge |  |
| 1-1-3 | learn about the humanities | □ Modify | □ Retain | □ Merge |  |
| 1-1-4 | learn about art | □ Modify | □ Retain | □ Merge |  |
| 1-2-1 | general ability | □ Modify | □ Retain | □ Merge |  |
| 1-2-2 | professional ability | □ Modify | □ Retain | □ Merge |  |
| 1-2-3 | career development ability | □ Modify | □ Retain | □ Merge |  |
| 2-1-1 | value | □ Modify | □ Retain | □ Merge |  |
| 2-2-1 | personal quality | ☑ Modify | □ Retain | □ Merge |  |
| 2-2-2 | professional quality | □ Modify | □ Retain | □ Merge |  |
| **Indexes that need to be added** | | | | | |
| **Add number** | **Index** | **Reasons for increasing the index** | | | |
|  |  |  | | | |
|  |  |  | | | |

4. Based on the Outcomes-Based Education (OBE) theory, student development theory, the needs of social and economic development, the goals and specifications of undergraduate vocational education talent training, and the results of previous research, this thesis develops specific 5^th^-level indexes for the development construct of students in undergraduate vocational universities. If you think this classification or expression is reasonable, please fill in the table below with your comments on modifications, retains, mergers, and additions.

| **Indexes that need perfect** | | | | | |
| --- | --- | --- | --- | --- | --- |
| **No.** | **Index** | **Disposal comments**  **(modify, retain, merge)** | | | **Expert comments and suggestions** |
| 1-1-1-1 | learn about science | □ Modify | □ Retain | □ Merge |  |
| 1-1-1-2 | learn about the humanities | □ Modify | □ Retain | □ Merge |  |
| 1-1-1-3 | learn about art | □ Modify | □ Retain | □ Merge |  |
| 1-1-1-4 | professional basic knowledge | □ Modify | □ Retain | □ Merge |  |
| 1-1-2-1 | deep professional theoretical knowledge | □ Modify | □ Retain | □ Merge |  |
| 1-1-2-2 | professional technical application knowledge | □ Modify | □ Retain | □ Merge |  |
| 1-1-2-3 | good oral presentation ability | □ Modify | □ Retain | □ Merge |  |
| 1-2-1-1 | well-written expression ability | □ Modify | □ Retain | □ Merge |  |
| 1-2-1-2 | foreign language application ability | □ Modify | □ Retain | □ Merge |  |
| 1-2-1-3 | proficiency in the application of information technology | ☑ Modify | □ Retain | □ Merge |  |
| 1-2-1-4 | organizational leadership ability | □ Modify | □ Retain | □ Merge |  |
| 1-2-1-5 | ability to cooperate effectively with others | □ Modify | □ Retain | □ Merge |  |
| 1-2-1-6 | self-learning ability | □ Modify | □ Retain | □ Merge |  |
| 1-2-1-7 | job adaptability | □ Modify | □ Retain | □ Merge |  |
| 1-2-2-1 | post operation ability | □ Modify | □ Retain | □ Merge |  |
| 1-2-2-2 | have the ability to solve problems on the job | □ Modify | □ Retain | □ Merge |  |
| 1-2-2-3 | possess the ability to innovate professional positions | □ Modify | □ Retain | □ Merge |  |
| 1-2-2-4 | emergency handling ability | □ Modify | □ Retain | □ Merge |  |
| 1-2-3-1 | career planning ability | □ Modify | □ Retain | □ Merge |  |
| 1-2-3-2 | career changeability | □ Modify | □ Retain | □ Merge |  |
| 1-2-3-3 | career mobility ability | □ Modify | □ Retain | □ Merge |  |
| 1-2-3-4 | career advancement ability | □ Modify | □ Retain | □ Merge |  |
| 1-3-1-1 | establishment of value | □ Modify | □ Retain | □ Merge |  |
| 1-3-1-2 | personal outlook on the world and life | □ Modify | □ Retain | □ Merge |  |
| 1-3-1-3 | understand the culture and value of different groups | □ Modify | □ Retain | □ Merge |  |
| 1-4-1-1 | self-awareness | □ Modify | □ Retain | □ Merge |  |
| 1-4-1-2 | personal character | □ Modify | □ Retain | □ Merge |  |
| 1-4-1-3 | physical and mental health | □ Modify | □ Retain | □ Merge |  |
| 1-4-1-4 | sense of responsibility | □ Modify | □ Retain | □ Merge |  |
| 1-4-2-1 | dialectical thinking | □ Modify | □ Retain | □ Merge |  |
| 1-4-2-2 | professional ethics | □ Modify | □ Retain | □ Merge |  |
| 1-4-2-3 | craftsman spirit | □ Modify | □ Retain | □ Merge |  |
| 1-4-2-4 | legal awareness | □ Modify | □ Retain | □ Merge |  |
| **Indexes that need to be added** | | | | | |
| **Add number** | **Index** | **Reasons for increasing the index** | | | |
|  |  |  | | | |
|  |  |  | | | |

Thank you again for your patience in reviewing and providing valuable comments!

|  |
| --- |
