# Supplementary material for: Constructing a student development model for undergraduate vocational universities in China using the Fuzzy Delphi Method and Analytic Hierarchy Process
Source: PLoS One. 2024 Mar 22;19(3):e0301017. doi: 10.1371/journal.pone.0301017 (PMC10959347; doi:10.1371/journal.pone.0301017)
Supplement: S1 Dataset — S1A, S1D and S1G are the first, second and third rounds of expert questionnaires respectively. S1C, S1E and S1H are the raw data of the first, second and third rounds of expert surveys respectively. S1B is the authoritative level data for the first round of expert surveys. S1F is the statistical analysis data of screening indicators in the second round of expert surveys. S1I is the statistical analysis data of indicator weight in the third round of expert surveys. (ZIP) [file pone.0301017.s001.zip › S1 Dataset/S1D Dataset (Second round of expert survey questionnaire).docx]

|  |
| --- |

**Expert Consultation Questionnaire on Student Development Construct Indexes in Undergraduate Vocational Universities-Screening Indexes**

Dear Experts，

Thank you very much for your help in completing this questionnaire out of your busy schedule. It is a questionnaire on screening student development construct indexes in undergraduate vocational education. To make the evaluation indexes identified in the thesis more objective and scientific, I hope to rely on your extensive experience and academic attainment in this field to provide valuable advice through this expert questionnaire. You are invited to evaluate the importance of each index, and your data will be processed to determine the screening of each index using the Fuzzy Delphi Technique (FDT). Your help will greatly assist the researcher in writing the thesis and is greatly appreciated. The information in your completed questionnaire is for academic research and will not be used for any other purpose.

**Personal information:**

Job title:

Position：

Years in current position：

Age：

Degree：

**Completion Instructions：**

This questionnaire aims to screen undergraduate vocational education student developmental construct indexes. The questionnaire is designed according to the form of the Fuzzy Delphi Technique. You are invited to evaluate the appropriateness and importance of each index by referring to the following evaluation guidelines. The evaluation is based on a scale of 1-9, with higher scores indicating more significant importance. Please rate the importance of each index according to your professionalism and fill in the integer values. The evaluation of each index consists of two parts.

Importance-evaluate: this index is vital to the upper level of indexes and enters a single integer value.

Acceptability-evaluate: this index’s acceptable range of importance to the upper level of indexes and enter the upper and lower acceptable values, respectively.

**Evaluation I**

Please complete the answer on the level of importance and acceptable range of the 2^nd^-lever indexes “cognitive and non-cognitive development” concerning the 1^st^-level index “student development”.

|  | **Level of importance** | **Acceptable range** | |
| --- | --- | --- | --- |
| **2^nd^-level index** | **Single value for your perceived level of importance (1-9)** | **The acceptable minimum value**  **(1-9)** | **The maximum acceptable value**  **(1-9)** |
| **student development (1^st^-level index)** | | | |
| cognitive development |  |  |  |
| non-cognitive development |  |  |  |

**EvaluationⅡ**

The 2^nd^-level indexes have sub-indexes, namely the 3^rd^-level indexes. Please fill in the answers on the importance of them to the 2^nd^-level indexes and their acceptable importance range.

|  | **Level of importance** | **Acceptable range** | |
| --- | --- | --- | --- |
| **3^rd^-level index** | **Single value for your perceived level of importance (1-9)** | **The acceptable minimum value**  **(1-9)** | **The maximum acceptable value**  **(1-9)** |
| **Cognitive development (2^nd^-level index**) | | | |
| knowledge development |  |  |  |
| ability development |  |  |  |
| **Non-cognitive development (2^nd^-level index**) | | | |
| quality development |  |  |  |

**Evaluation Ⅲ**

The 3^rd^-level indexes have sub-indexes, namely the 4^th^-level indexes. Please fill in the answers on the importance of them to the 3^rd^-level indexes and their acceptable importance range.

|  | **Level of importance** | **Acceptable range** | | |
| --- | --- | --- | --- | --- |
| **4^th^-level index** | **Single value for your perceived level of importance (1-9)** | **The acceptable minimum value**  **(1-9)** | **The maximum acceptable value**  **(1-9)** | |
| **Knowledge development (3**^rd^**-level index**) | | | | |
| general knowledge |  |  | |  |
| professional knowledge |  |  | |  |
| **Ability development (3^rd^-level index**) | | | | |
| general ability |  |  | |  |
| professional ability |  |  | |  |
| career development ability |  |  | |  |
| **Quality development (3^rd^-level index**) | | | | |
| value |  |  | |  |
| personal quality |  |  | |  |
| professional quality |  |  | |  |

**Evaluation Ⅳ**

The 4^th^-level indexes have sub-indexes, namely the 5^th^-level indexes. Please fill in the answers as above.

|  | **Level of importance** | **Acceptable range** | |
| --- | --- | --- | --- |
| **5^th^-level index** | **Single value for your perceived level of importance (1-9)** | **The acceptable minimum value (1-9)** | **The maximum acceptable value (1-9)** |
| **General knowledge (4^th^-level index)** | | | |
| learn about science |  |  |  |
| learn about the humanities |  |  |  |
| learn about art |  |  |  |
| **Professional knowledge（(4^th^-level index)** | | | |
| professional basic knowledge |  |  |  |
| deep professional theoretical knowledge |  |  |  |
| professional technical application knowledge |  |  |  |
| **General ability(4^th^ -level index)** | | | |
| good oral presentation ability |  |  |  |
| well-written expression ability |  |  |  |
| foreign language application ability |  |  |  |
| proficiency in the application of information technology |  |  |  |
| organizational leadership ability |  |  |  |
| ability to cooperate effectively with others |  |  |  |
| self-learning ability |  |  |  |
| **Professional ability(4^th^-level index)** | | | |
| job adaptability |  |  |  |
| post operation ability |  |  |  |
| ability to solve problems on the job |  |  |  |
| ability to innovate professional positions |  |  |  |
| emergency handling ability |  |  |  |
| **Career development ability(4^th^-level index)** | | | |
| career planning ability |  |  |  |
| career changeability |  |  |  |
| career mobility ability |  |  |  |
| career advancement ability |  |  |  |
| **Value(4^th^-level index)** | | | |
| establishment of value |  |  |  |
| personal outlook on the world and life |  |  |  |
| understanding of the culture and values of different groups |  |  |  |
| **Personal quality(4^th^-level index)** | | | |
| self-awareness |  |  |  |
| personal character |  |  |  |
| physical and mental health |  |  |  |
| sense of responsibility |  |  |  |
| dialectical thinking |  |  |  |
| **Professional quality(4^th^-level index)** | | | |
| professional ethics |  |  |  |
| craftsman spirit |  |  |  |
| legal awareness |  |  |  |
