# Supplementary material for: Constructing a student development model for undergraduate vocational universities in China using the Fuzzy Delphi Method and Analytic Hierarchy Process
Source: PLoS One. 2024 Mar 22;19(3):e0301017. doi: 10.1371/journal.pone.0301017 (PMC10959347; doi:10.1371/journal.pone.0301017)
Supplement: S1 Dataset — S1A, S1D and S1G are the first, second and third rounds of expert questionnaires respectively. S1C, S1E and S1H are the raw data of the first, second and third rounds of expert surveys respectively. S1B is the authoritative level data for the first round of expert surveys. S1F is the statistical analysis data of screening indicators in the second round of expert surveys. S1I is the statistical analysis data of indicator weight in the third round of expert surveys. (ZIP) [file pone.0301017.s001.zip › S1 Dataset/S1G Dataset (Third round of expert survey questionnaire).docx]

|  |
| --- |

**Expert Consultation Questionnaire on Student Development Construct Indexes in Undergraduate Vocational Universities**

**-Weight Survey**

Dear experts,

This questionnaire determines the weight of each index in the student development construct in undergraduate vocational education. The data collected were analyzed using Analytic Hierarchy Process. The hierarchical construct of student development in undergraduate vocational education is presented in the table below.

**The hierarchical construct of undergraduate vocational education student development：**

| **Goal**  **(1^st^-level index)** | **Element**  **(2^nd^-level index)** | **Dimension**  **(3^rd^-level index)** | **Factor**  **(4^th^-level index)** | **Program**  **(5^th^-level index)** |
| --- | --- | --- | --- | --- |
| student  development | cognitive development  (B1) | knowledge  development  (C1) | general  knowledge  (D1) | learn about science (E1) |
|  |  |  |  | learn about the humanities (E2) |
|  |  |  |  | learn about art (E3) |
|  |  |  | professional knowledge  (D2) | professional basic knowledge (E4) |
|  |  |  |  | deep professional theoretical knowledge (E5) |
|  |  |  |  | professional technical application knowledge (E6) |
|  |  | ability development  (C2) | general ability  (D3) | good oral presentation ability (E7) |
|  |  |  |  | well-written expression ability (E8) |
|  |  |  |  | foreign language application ability (E9) |
|  |  |  |  | proficiency in the application of information technology (E10) |
|  |  |  |  | organizational leadership ability (E11) |
|  |  |  |  | ability to cooperate effectively with others (E12) |
|  |  |  |  | self-learning ability (E13) |
|  |  |  | professional ability  (D4) | job adaptability (E14) |
|  |  |  |  | post operation ability (E15) |
|  |  |  |  | have the ability to solve problems on the job (E16) |
|  |  |  |  | possess the ability to innovate professional positions (E17) |
|  |  |  |  | emergency handling ability (E18) |
|  |  |  | career development ability (D5) | career planning ability (E19) |
|  |  |  |  | career changeability (E20) |
|  |  |  |  | career mobility ability (E21) |
|  |  |  |  | career advancement ability (E22) |
|  | non-  cognitive development  (B2) | quality  development  (C3) | value (D6) | establishment of value (E23) |
|  |  |  |  | personal outlook on the world and life (E24) |
|  |  |  |  | understand the culture and values of different groups (E25) |
|  |  |  | personal quality  (D7) | self-awareness (E26) |
|  |  |  |  | personal character (E27) |
|  |  |  |  | physical and mental health (E28) |
|  |  |  |  | sense of responsibility (E29) |
|  |  |  |  | dialectical thinking (E30) |
|  |  |  | professional quality  (D8) | professional ethics (E31) |
|  |  |  |  | craftsman spirit (E32) |
|  |  |  |  | legal awareness (E33) |

To make the student development construct indexes proposed in this thesis more objective and scientific, we hope to rely on your extensive experience and academic attainment in this field to provide valuable comments through this expert questionnaire. By comparing the importance of each index to the objective, you will be able to determine the weight of each index. Your help will greatly assist the researcher in writing her thesis and is greatly appreciated. The information you provide in the questionnaire is for academic research and will not be used for any other purpose.

**Personal information:**

Job title:

Position：

Years in current position：

Age：

Degree：

**Completion instructions:**

This questionnaire is an Analytic Hierarchy Process questionnaire. This method compares the importance of two indexes at the same level against the upper level. Please rank the indexes in order of importance as listed, then compare each two indexes later to select their significance.

**Part I Ranking the importance of 2^nd^-level indexes**

**1. Importance ranking**

For student development indexes, the relative importance of **(1) cognitive development** and **(2) non-cognitive development**:

Ranking of the degree of importance of the two indexes: ( ) ≥ ( ) (please fill in the numbers).

**2. Every two indexes comparative evaluation**

Absolutely important (9), very important (7), relatively important (5), slightly important (3), equally important (1), 8, 6, 4, 2, indicate the middle of two critical levels.

Measures to the left index that the indexes in the left column are more important than those in the right column, and those to the right index that the indexes in the right column are more important than those in the left column. Tick “√” the corresponding box according to your opinion.

**Example:** If you think that index A, “Cognitive development,” on the left is absolutely important compared to index B, “Non-cognitive development,” on the right, then mark “√” in column 9 on the left. If you think that “Cognitive development” should be significant compared to “Non-cognitive development”, then you can mark “√” in column 9 on the right

| **Index A** | **Left side is absolutely important** |  | **Left side is very important** |  | **Left side is more important** |  | **Left side is slightly important** |  | **Both are equally important** |  | **Right side is slightly important** |  | **Right side is more important** |  | **Right side is very important** |  | **Right side is absolutely important** | **Index B** |
| --- | --- | --- | --- | --- | --- | --- | --- | --- | --- | --- | --- | --- | --- | --- | --- | --- | --- | --- |
|  | 9 | 8 | 7 | 6 | 5 | 4 | 3 | 2 | 1 | 2 | 3 | 4 | 5 | 6 | 7 | 8 | 9 |  |
| cognitive development |  |  |  |  |  |  |  |  |  |  |  |  |  |  |  |  |  | non-cognitive development |

**PartⅡ Ranking the importance of 3^rd^-level indexes**

**1. Importance ranking**

For student development indexes, the relative importance of **(1) knowledge development** and **(2) ability development:**

Ranking of the degree of importance of the two indexes: ( ) ≥ ( ) (please fill in the numbers).

**2. Every two indexes comparative evaluation**

| **Index A** | **Left side is absolutely important** |  | **Left side is very important** |  | **Left side is more important** |  | **Left side is slightly important** |  | **Both are equally important** |  | **Right side is slightly important** |  | **Right side is more important** |  | **Right side is very important** |  | **Right side is absolutely important** | **Index B** |
| --- | --- | --- | --- | --- | --- | --- | --- | --- | --- | --- | --- | --- | --- | --- | --- | --- | --- | --- |
|  | 9 | 8 | 7 | 6 | 5 | 4 | 3 | 2 | 1 | 2 | 3 | 4 | 5 | 6 | 7 | 8 | 9 |  |
| knowledge development |  |  |  |  |  |  |  |  |  |  |  |  |  |  |  |  |  | ability development |

**Part Ⅲ Ranking the importance of 4^th^-level indexes**

**1. Knowledge development aspect**

**Importance ranking**. For knowledge development, the relative importance of the indexes of **(1) general knowledge and (2) professional knowledge.**

Ranking of the degree of importance of the two indexes: ( ) ≥ ( ) (please fill in the numbers).

**Each two indexes comparative evaluation:**

| **Index A** | **Left side is absolutely important** |  | **Left side is very important** |  | **Left side is more important** |  | **Left side is slightly important** |  | **Both are equally important** |  | **Right side is slightly important** |  | **Right side is more important** |  | **Right side is very important** |  | **Right side is absolutely important** | **Index B** |
| --- | --- | --- | --- | --- | --- | --- | --- | --- | --- | --- | --- | --- | --- | --- | --- | --- | --- | --- |
|  | 9 | 8 | 7 | 6 | 5 | 4 | 3 | 2 | 1 | 2 | 3 | 4 | 5 | 6 | 7 | 8 | 9 |  |
| general knowledge |  |  |  |  |  |  |  |  |  |  |  |  |  |  |  |  |  | professional knowledge |

**2. Ability development aspect**

**Importance ranking**. For ability development, the relative degree of importance of the indexes of **(1) general ability**, **(2) professional ability,** and **(3) career development ability**:

Ranking of the degree of importance of the three indexes: ( ) ≥ ( ) ≥ ( ) (please fill in the numbers).

**Every two indexes comparative evaluation:**

| **Index A** | **Left side is absolutely important** |  | **Left side is very important** |  | **Left side is more important** |  | **Left side is slightly important** |  | **Both are equally important** |  | **Right side is slightly important** |  | **Right side is more important** |  | **Right side is very important** |  | **Right side is absolutely important** | **Index B** |
| --- | --- | --- | --- | --- | --- | --- | --- | --- | --- | --- | --- | --- | --- | --- | --- | --- | --- | --- |
|  | 9 | 8 | 7 | 6 | 5 | 4 | 3 | 2 | 1 | 2 | 3 | 4 | 5 | 6 | 7 | 8 | 9 |  |
| general ability |  |  |  |  |  |  |  |  |  |  |  |  |  |  |  |  |  | professional knowledge |
| general ability |  |  |  |  |  |  |  |  |  |  |  |  |  |  |  |  |  | career development ability |
| professional ability |  |  |  |  |  |  |  |  |  |  |  |  |  |  |  |  |  | career development ability |

**3. Quality development aspect**

**Importance ranking**. For quality development, the relative degree of importance of indexes of **(1) value, (2) personal quality, and (3) professional quality**:

Ranking of the degree of importance of the three indexes: ( ) ≥ ( ) ≥ ( ) (please fill in the numbers)

**Every two indexes comparative evaluation:**

| **Index A** | **Left side is absolutely important** |  | **Left side is very important** |  | **Left side is more important** |  | **Left side is slightly important** |  | **Both are equally important** |  | **Right side is slightly important** |  | **Right side is more important** |  | **Right side is very important** |  | **Right side is absolutely important** | **Index B** |
| --- | --- | --- | --- | --- | --- | --- | --- | --- | --- | --- | --- | --- | --- | --- | --- | --- | --- | --- |
|  | 9 | 8 | 7 | 6 | 5 | 4 | 3 | 2 | 1 | 2 | 3 | 4 | 5 | 6 | 7 | 8 | 9 |  |
| value |  |  |  |  |  |  |  |  |  |  |  |  |  |  |  |  |  | personal quality |
| value |  |  |  |  |  |  |  |  |  |  |  |  |  |  |  |  |  | professional quality |
| personal quality |  |  |  |  |  |  |  |  |  |  |  |  |  |  |  |  |  | professional quality |
