# Supplementary material for: Constructing a student development model for undergraduate vocational universities in China using the Fuzzy Delphi Method and Analytic Hierarchy Process
Source: PLoS One. 2024 Mar 22;19(3):e0301017. doi: 10.1371/journal.pone.0301017 (PMC10959347; doi:10.1371/journal.pone.0301017)
Supplement: S1 Dataset — S1A, S1D and S1G are the first, second and third rounds of expert questionnaires respectively. S1C, S1E and S1H are the raw data of the first, second and third rounds of expert surveys respectively. S1B is the authoritative level data for the first round of expert surveys. S1F is the statistical analysis data of screening indicators in the second round of expert surveys. S1I is the statistical analysis data of indicator weight in the third round of expert surveys. (ZIP) [file pone.0301017.s001.zip › S1 Dataset/S1I Dataset (Statistical analysis data of indicator weight in the third round of expert surveys).docx]

**Final indicator weight and ranking.**

| **1^st^-level indicator** | **2^nd^-level indicator** | **Weight** | **3^rd^-level indicator** | **Relative weight** | **Absolute weight** | **Ranking** | **4^th^-level indicator** | **Relative weight** | **Absolute weight** | **Ranking** |
| --- | --- | --- | --- | --- | --- | --- | --- | --- | --- | --- |
| Student development (A) | Cognition  （B1） | 0.50 | Knowledge  （C1） | 0.33 | 0.17 | 3 | General knowledge (D1) | 0.37 | 0.06 | 7 |
|  |  |  |  |  |  |  | Professional knowledge (D2) | 0.63 | 0.10 | 6 |
|  |  |  | Ability  （C2） | 0.67 | 0.33 | 2 | General ability (D3) | 0.11 | 0.04 | 8 |
|  |  |  |  |  |  |  | Professional ability (D4) | 0.32 | 0.11 | 5 |
|  |  |  |  |  |  |  | Career development ability (D5) | 0.57 | 0.19 | 2 |
|  | Non-  cognition  （B2） | 0.50 | Quality  （C3） | 1 | 0.50 | 1 | Value (D6) | 0.47 | 0.24 | 1 |
|  |  |  |  |  |  |  | Personal quality (D7) | 0.28 | 0.14 | 3 |
|  |  |  |  |  |  |  | Professional quality (D8) | 0.25 | 0.16 | 4 |
