# Supplementary material for: Constructing a student development model for undergraduate vocational universities in China using the Fuzzy Delphi Method and Analytic Hierarchy Process
Source: PLoS One. 2024 Mar 22;19(3):e0301017. doi: 10.1371/journal.pone.0301017 (PMC10959347; doi:10.1371/journal.pone.0301017)
Supplement: S1 Dataset — S1A, S1D and S1G are the first, second and third rounds of expert questionnaires respectively. S1C, S1E and S1H are the raw data of the first, second and third rounds of expert surveys respectively. S1B is the authoritative level data for the first round of expert surveys. S1F is the statistical analysis data of screening indicators in the second round of expert surveys. S1I is the statistical analysis data of indicator weight in the third round of expert surveys. (ZIP) [file pone.0301017.s001.zip › S1 Dataset/S1B Dataset (Authoritative level data from the first round of expert surveys).doc]

**Expert Authority Level**

| **Expert number** | **Familiarity coefficient**   | **Judgment basis** | | | | **Judgment basis**   | **Authority coefficient**  |
| --- | --- | --- | --- | --- | --- | --- | --- |
|  |  | **Practical experience** | **Logical reasoning** | **Knowledge of domestic and international** | **Intuition** |  |  |
| 1 | 0.75 | 0.50 | 0.30 | 0.10 | 0.10 | 1.00 | 0.88 |
| 2 | 1.00 | 0.50 | 0.30 | 0.10 | 0.10 | 1.00 | 1.00 |
| 3 | 0.75 | 0.50 | 0.20 | 0.10 | 0.10 | 0.90 | 0.83 |
| 4 | 0.50 | 0.40 | 0.30 | 0.10 | 0.10 | 0.90 | 0.70 |
| 5 | 0.75 | 0.50 | 0.20 | 0.10 | 0.10 | 0.90 | 0.83 |
| 6 | 0.75 | 0.50 | 0.30 | 0.10 | 0.10 | 1.00 | 0.88 |
| 7 | 1.00 | 0.50 | 0.20 | 0.10 | 0.10 | 0.90 | 0.95 |
| 8 | 1.00 | 0.50 | 0.30 | 0.10 | 0.10 | 1.00 | 1.00 |
| 9 | 0.75 | 0.50 | 0.30 | 0.10 | 0.10 | 1.00 | 0.88 |
| 10 | 0.75 | 0.50 | 0.30 | 0.10 | 0.10 | 1.00 | 0.88 |
| 11 | 1.00 | 0.50 | 0.30 | 0.10 | 0.10 | 1.00 | 1.00 |
| 12 | 0.75 | 0.50 | 0.30 | 0.10 | 0.10 | 1.00 | 0.88 |
| 13 | 0.75 | 0.40 | 0.30 | 0.10 | 0.10 | 0.90 | 0.83 |
| 14 | 1.00 | 0.50 | 0.30 | 0.10 | 0.10 | 1.00 | 1.00 |
| 15 | 1.00 | 0.50 | 0.30 | 0.10 | 0.10 | 1.00 | 1.00 |
| 16 | 0.75 | 0.50 | 0.20 | 0.10 | 0.10 | 0.90 | 0.83 |
| 17 | 1.00 | 0.50 | 0.30 | 0.10 | 0.10 | 1.00 | 1.00 |
| 18 | 0.75 | 0.50 | 0.30 | 0.10 | 0.10 | 1.00 | 0.88 |
| 19 | 1.00 | 0.50 | 0.30 | 0.10 | 0.10 | 1.00 | 1.00 |
| 20 | 0.75 | 0.50 | 0.20 | 0.10 | 0.10 | 0.90 | 0.83 |
| Mean value | 0.84 | 0.49 | 0.28 | 0.10 | 0.10 | 0.97 | 0.91 |
