# Supplementary material for: Constructing a student development model for undergraduate vocational universities in China using the Fuzzy Delphi Method and Analytic Hierarchy Process
Source: PLoS One. 2024 Mar 22;19(3):e0301017. doi: 10.1371/journal.pone.0301017 (PMC10959347; doi:10.1371/journal.pone.0301017)
Supplement: S1 Dataset — S1A, S1D and S1G are the first, second and third rounds of expert questionnaires respectively. S1C, S1E and S1H are the raw data of the first, second and third rounds of expert surveys respectively. S1B is the authoritative level data for the first round of expert surveys. S1F is the statistical analysis data of screening indicators in the second round of expert surveys. S1I is the statistical analysis data of indicator weight in the third round of expert surveys. (ZIP) [file pone.0301017.s001.zip › S1 Dataset/S1C Dataset (Raw data from the first round of expert surveys).doc]

**~~Results of the first round of the expert survey.~~**

| **Indicators to be refined** | | | |
| --- | --- | --- | --- |
| **Original No.** | **Indicator** | **Expert Suggestion** | **Handling Opinion** |
| C3 | value | Value is part of quality. It is recommended to merge value and quality. | Merge C3 with C4. |
| C4 | quality | Quality covers value. It is proposed to merge the two. | Merge C4 with C3. |
| E6 | deep professional theoretical knowledge | Undergraduate vocational education emphasizes technical theory. Modify it to deep professional theoretical knowledge. | Modify E6. |
| E12 | organizational leadership ability | Students work in technical skills, and enterprises do not demand high organizational leadership. So, modify it to some organizational leadership. | Modify E12. |
| **Indicators to be added** | | | |
| **Original No.** | **Indicators** | **Reasons for adding indicators** | **Handling Opinion** |
| E19 | emergency handling ability | Students on the production line must possess excellent emergency handling ability to reduce accident damage. | The vocational ability module should include emergency handling ability. |
| E30 | dialectical thinking | Students need to think dialectically and view technical skills jobs with a developmental eye. | The personal quality module should add dialectical thinking. |
| E34 | legal awareness | Students must have rigorous legal awareness throughout their careers. | The vocational quality module needs to add legal awareness. |
