# Supplementary material for: Constructing a student development model for undergraduate vocational universities in China using the Fuzzy Delphi Method and Analytic Hierarchy Process
Source: PLoS One. 2024 Mar 22;19(3):e0301017. doi: 10.1371/journal.pone.0301017 (PMC10959347; doi:10.1371/journal.pone.0301017)
Supplement: S1 Dataset — S1A, S1D and S1G are the first, second and third rounds of expert questionnaires respectively. S1C, S1E and S1H are the raw data of the first, second and third rounds of expert surveys respectively. S1B is the authoritative level data for the first round of expert surveys. S1F is the statistical analysis data of screening indicators in the second round of expert surveys. S1I is the statistical analysis data of indicator weight in the third round of expert surveys. (ZIP) [file pone.0301017.s001.zip › S1 Dataset/S1F Dataset (Statistical analysis data of ccreening indicators in the second round of expert surveys).doc]

**Statistical Analysis Results of Screening of Student Development Model Indicators**

| **5^th^-level index** | **The lower limit of acceptable range** | | **The upper limit of the acceptable range** | | **A single value of importance**  | | **Geometric mean** | | | **A grey area** | **Convergence test** | **A grey area test value** | **Importance value** | **Processing** |
| --- | --- | --- | --- | --- | --- | --- | --- | --- | --- | --- | --- | --- | --- | --- |
|  |  |  |  |  |  |  |  |  |  |  |  |  |  |  |
| E1 | 1 | 2 | 1 | 5 | 1 | 3 | 1.40 | 3.00 | 1 | 1 | 1.60 | 0.60 | 2.12 | Delete |
| E2 | 5 | 8 | 7 | 9 | 6 | 9 | 6.55 | 8.65 | 7 | 1 | 2.10 | 1.10 | 7.70 |  |
| E3 | 6 | 8 | 7 | 9 | 7 | 9 | 6.55 | 8.65 | 6 | 1 | 2.10 | 1.10 | 7.68 |  |
| E4 | 5 | 8 | 7 | 9 | 6 | 9 | 6.30 | 8.80 | 6 | 1 | 2.50 | 1.50 | 7.67 |  |
| E5 | 5 | 8 | 7 | 9 | 6 | 9 | 6.60 | 8.65 | 5 | 1 | 2.05 | 1.05 | 7.65 |  |
| E6 | 6 | 8 | 7 | 9 | 7 | 9 | 6.65 | 8.90 | 6 | 1 | 2.25 | 1.25 | 7.93 |  |
| E7 | 6 | 8 | 7 | 9 | 7 | 9 | 6.65 | 8.90 | 6 | 1 | 2.25 | 1.25 | 7.93 |  |
| E8 | 6 | 9 | 8 | 9 | 7 | 9 | 6.70 | 8.90 | 6 | 1 | 2.20 | 1.20 | 7.95 |  |
| E9 | 5 | 8 | 7 | 9 | 6 | 9 | 6.60 | 8.60 | 7 | 1 | 2.00 | 1.00 | 7.70 |  |
| E10 | 5 | 8 | 7 | 9 | 6 | 9 | 6.80 | 8.65 | 5 | 1 | 1.85 | 0.85 | 7.72 |  |
| E11 | 6 | 8 | 7 | 9 | 7 | 9 | 6.60 | 8.85 | 6 | 1 | 2.25 | 1.25 | 7.82 |  |
| E12 | 6 | 8 | 7 | 9 | 7 | 9 | 6.60 | 8.65 | 6 | 1 | 2.05 | 1.05 | 7.70 |  |
| E13 | 6 | 9 | 7 | 9 | 7 | 9 | 6.75 | 8.80 | 6 | 2 | 2.05 | 0.05 | 7.92 |  |
| E14 | 6 | 8 | 7 | 9 | 7 | 9 | 6.75 | 8.90 | 6 | 1 | 2.15 | 1.15 | 8.05 |  |
| E15 | 6 | 8 | 7 | 9 | 7 | 9 | 6.60 | 8.80 | 6 | 1 | 2.20 | 1.20 | 7.95 |  |
| E16 | 6 | 8 | 7 | 9 | 7 | 9 | 6.85 | 8.85 | 6 | 1 | 2.00 | 1.00 | 8.03 |  |
| E17 | 6 | 8 | 7 | 9 | 8 | 9 | 6.85 | 8.90 | 6 | 1 | 2.05 | 1.05 | 8.15 |  |
| E18 | 6 | 8 | 7 | 9 | 7 | 9 | 6.60 | 8.70 | 6 | 1 | 2.10 | 1.10 | 7.83 |  |
| E19 | 6 | 8 | 7 | 9 | 7 | 9 | 6.70 | 8.85 | 6 | 1 | 2.15 | 1.15 | 7.97 |  |
| E20 | 6 | 8 | 7 | 9 | 7 | 9 | 6.50 | 8.70 | 6 | 1 | 2.20 | 1.20 | 7.73 |  |
| E21 | 6 | 8 | 7 | 9 | 7 | 9 | 6.55 | 8.75 | 6 | 1 | 2.20 | 1.20 | 7.72 |  |
| E22 | 6 | 9 | 8 | 9 | 6 | 9 | 6.95 | 8.95 | 6 | 1 | 2.00 | 1.00 | 8.02 |  |
| E23 | 5 | 8 | 7 | 9 | 7 | 9 | 6.90 | 8.85 | 6 | 1 | 1.95 | 0.95 | 7.87 |  |
| E24 | 6 | 9 | 8 | 9 | 8 | 9 | 7.10 | 8.95 | 6 | 1 | 1.85 | 0.85 | 8.28 |  |
| E25 | 6 | 9 | 8 | 9 | 8 | 9 | 6.90 | 8.95 | 6 | 1 | 2.05 | 1.05 | 8.15 |  |
| E26 | 6 | 8 | 7 | 9 | 7 | 9 | 6.55 | 8.70 | 6 | 1 | 2.15 | 1.15 | 7.70 |  |
| E27 | 6 | 8 | 7 | 9 | 7 | 9 | 6.85 | 8.90 | 6 | 1 | 2.05 | 1.05 | 8.07 |  |
| E28 | 6 | 8 | 7 | 9 | 7 | 9 | 6.95 | 8.90 | 6 | 1 | 1.95 | 0.95 | 8.20 |  |
| E29 | 6 | 9 | 8 | 9 | 8 | 9 | 6.90 | 8.95 | 6 | 1 | 2.05 | 1.05 | 8.20 |  |
| E30 | 6 | 9 | 8 | 9 | 8 | 9 | 6.95 | 8.95 | 6 | 1 | 2.00 | 1.00 | 8.23 |  |
| E31 | 3 | 8 | 7 | 9 | 7 | 9 | 6.35 | 8.85 | 6 | 1 | 2.50 | 1.50 | 7.68 |  |
| E32 | 6 | 9 | 8 | 9 | 8 | 9 | 6.85 | 8.95 | 6 | 1 | 2.10 | 1.10 | 8.18 |  |
| E33 | 2 | 6 | 4 | 9 | 3 | 9 | 2.90 | 5.00 | 2 | 2 | 2.10 | 0.10 | 3.90 | Delete |
| E34 | 1 | 8 | 4 | 9 | 1 | 3 | 2.20 | 8.10 | 2 | 4 | 5.90 | 1.90 | 4.23 | Delete |
| E35 | 2 | 8 | 7 | 9 | 4 | 9 | 6.30 | 8.90 | 6 | 1 | 2.60 | 1.60 | 7.70 |  |
| E36 | 6 | 9 | 8 | 9 | 7 | 9 | 6.75 | 8.95 | 6 | 1 | 2.20 | 1.20 | 8.03 |  |
