# Supplementary material for: Constructing a student development model for undergraduate vocational universities in China using the Fuzzy Delphi Method and Analytic Hierarchy Process
Source: PLoS One. 2024 Mar 22;19(3):e0301017. doi: 10.1371/journal.pone.0301017 (PMC10959347; doi:10.1371/journal.pone.0301017)
Supplement: S1 Dataset — S1A, S1D and S1G are the first, second and third rounds of expert questionnaires respectively. S1C, S1E and S1H are the raw data of the first, second and third rounds of expert surveys respectively. S1B is the authoritative level data for the first round of expert surveys. S1F is the statistical analysis data of screening indicators in the second round of expert surveys. S1I is the statistical analysis data of indicator weight in the third round of expert surveys. (ZIP) [file pone.0301017.s001.zip › S1 Dataset/S1H Dataset (Raw data from the third round of expert surveys).doc]

| **Raw data from the second round of expert surveys** | | | | | | | | | |
| --- | --- | --- | --- | --- | --- | --- | --- | --- | --- |
| **Expert No.** | **B1/B2** | **C1/C2** | **D1/D2** | **D3/D4** | **D3/D5** | **D4/D5** | **D6/D7** | **D6/D8** | **D7/D8** |
| Expert 1 | 1/2 | 2/1 | 1/2 | 1/3 | 1/5 | 1/2 | 2/1 | 2/1 | 1/1 |
| Expert 2 | 1/1 | 1/4 | 1/4 | 1/7 | 1/7 | 1/1 | 5/1 | 3/1 | 1/1 |
| Expert 3 | 1/2 | 1/2 | 1/3 | 1/3 | 1/9 | 1/3 | 3/1 | 4/1 | 2/1 |
| Expert 4 | 3/1 | 1/1 | 1/2 | 1/4 | 1/9 | 4/9 | 1/1 | 1/1 | 1/1 |
| Expert 5 | 1/1 | 1/3 | 1/5 | 2/7 | 1/4 | 6/7 | 2/1 | 9/1 | 2/1 |
| Expert 6 | 1/1 | 1/3 | 1/1 | 2/1 | 1/2 | 1/4 | 3/1 | 5/1 | 2/1 |
| Expert 7 | 1/1 | 1/3 | 1/4 | 5/8 | 2/7 | 3/7 | 1/1 | 1/1 | 1/1 |
| Expert 8 | 1/3 | 1/2 | 1/2 | 2/7 | 1/8 | 4/9 | 2/1 | 9/1 | 2/1 |
| Expert 9 | 2/1 | 1/5 | 1/3 | 3/8 | 2/9 | 4/7 | 3/1 | 4/1 | 2/1 |
| Expert 10 | 3/1 | 1/1 | 1/2 | 5/9 | 2/7 | 5/9 | 9/1 | 3/1 | 3/1 |
| Expert 11 | 1/1 | 1/2 | 1/2 | 1/3 | 1/7 | 1/2 | 1/1 | 1/1 | 1/1 |
| Expert 12 | 1/3 | 1/4 | 1/4 | 3/8 | 2/9 | 4/7 | 3/1 | 2/1 | 3/1 |
| Expert 13 | 2/1 | 1/2 | 1/1 | 3/8 | 2/9 | 4/7 | 3/1 | 4/1 | 2/1 |
| Expert 14 | 1/1 | 1/2 | 1/3 | 3/8 | 1/5 | 5/9 | 2/1 | 2/1 | 1/1 |
| Expert 15 | 1/1 | 1/1 | 1/3 | 1/4 | 1/9 | 4/9 | 1/1 | 2/1 | 3/1 |
| Expert 16 | 1/1 | 1/3 | 1/1 | 2/7 | 1/4 | 6/7 | 1/1 | 1/1 | 1/1 |
| Expert 17 | 2/1 | 1/2 | 1/3 | 1/7 | 1/7 | 1/1 | 2/1 | 2/1 | 1/1 |
| Expert 18 | 1/1 | 1/3 | 1/1 | 3/8 | 1/5 | 5/9 | 1/1 | 3/1 | 3/1 |
| Expert 19 | 1/3 | 1/2 | 1/3 | 3/8 | 1/5 | 5/9 | 2/1 | 9/1 | 2/1 |
| Expert 20 | 1/1 | 1/2 | 1/2 | 3/8 | 2/9 | 4/7 | 1/1 | 1/1 | 1/1 |
